# High serum adiponectin as a biomarker of renal dysfunction: Results from the KNOW-CKD study

**DOI:** 10.1038/s41598-020-62465-2

**Published:** 2020-03-27

**Authors:** Su Hyun Song, Tae Ryom Oh, Hong Sang Choi, Chang Seong Kim, Seong Kwon Ma, Kook Hwan Oh, Curie Ahn, Soo Wan Kim, Eun Hui Bae

**Affiliations:** 1 Department of Internal Medicine, Chonnam National University Medical School, Gwangju, Korea; 2 Department of Internal Medicine, Seoul National University Hospital, Seoul, Korea

**Keywords:** Chronic kidney disease, Prognostic markers

## Abstract

High serum adiponectin is noted in several conditions of chronic kidney disease (CKD) and is a predictor for end stage renal disease. However, the relationship between adiponectin level and renal disease progression is not well established. This study aimed to determine the relationship between serum adiponectin levels and CKD progression. This prospective longitudinal study included 2238 patients from the Korean Cohort Study for Outcomes in Patients with Chronic Kidney Disease. Patients were divided into quartiles according to their serum adiponectin level. Composite renal outcome was defined as one or more of the following: initiation of dialysis or transplantation, a two-fold increase in baseline serum creatinine levels, or a 50% decline in the estimated glomerular filtration rate (eGFR) during the follow-up period. A cox proportional hazard ratio model was applied to analyze the relationship between composite renal outcome and serum adiponectin levels. Serum adiponectin level was inversely associated with eGFR (p < 0.001) and positively correlated with urine albumin-creatinine ratio. The highest quartile of serum adiponectin was associated with an increased risk of adverse renal outomes (HR, 1.39; 95%CI, 1.05-1.84; p=0.021). On time-dependent receiver operating characteristic curve analysis, predictive ability of adiponectin for renal outcomes disappeared after adjusting for eGFR. Therefore, serum adiponectin may be a biomarker of renal dysfunction rather than a true risk factor in CKD progression.

## Introduction

Chronic kidney disease (CKD) is a major public burden, with rising global prevalence^1^. There is an urgent need to identify factors that may predict and prevent CKD progression.

Adiponectin is a peptide hormone secreted from adipocytes, with anti-inflammatory, anti-diabetic, and anti-atherogenic properties^2,3^. Low serum levels of adiponectin have been associated with obesity, insulin resistance, coronary heart disease, and metabolic syndrome in the general population^4–6^. Paradoxically, serum adiponectin levels are elevated in patients with CKD and end-stage renal disease (ESRD)^7^. Moreover, high serum adiponectin levels were associated with increased mortality in patients with stage 3 or 4 CKD in the Modification of Diet in Renal Disease (MDRD) study^8^. High serum adiponectin predicts all-cause mortality and ESRD in type 1 diabetics^9,10^ and is associated with increased albuminuria in CKD patients^11^. High adiponectin has been found to predict renal disease progression in men with CKD, but not in women^12^. Adiponectin has also been markedly increased in patients with nephrotic syndrome^13^ and essential hypertension^14^.

However, in a study, transgenic upregulation of adiponectin in CKD mice prevented renal injury^15^. Hypoadiponectinemia is also independently associated with the presence of metabolic syndrome, which is a risk factor for CKD^16^. In addition, most research regarding adiponectin has been conducted in relatively small populations with a limited number of patients with type 1 diabetes^9^ or obesity^17^. Thus, the association between serum adiponectin and renal disease outcome remains controversial. We aimed to determine the relationship between serum adiponectin levels and renal outcomes in a large Korean pre-dialysis CKD cohort.

## Results

### Baseline characteristics

Baseline characteristics of patients are presented in Table 1, along with their serum adiponectin quartiles. The mean age was 53.5 ± 12.2 years, and 814 (36.4%) of patients were women. The mean eGFR was 53.1 ± 30.8 ml/min/1.73 m^2^, and the mean serum adiponectin level was 12.2 μg/mL. The mean values for each serum adiponectin quartile were 2.9 ± 1.4, 7.2 ± 1.2, 12.7 ± 2.0, and 26.1 ± 9.6 μg/mL, respectively.

**Table 1:** Baseline characteristics of the patients according to serum adiponectin quartiles.

Parameters	Serum adiponectin quartiles				p-value
	Quartile 1 (n = 525) (≤5.09 *μ*g/ml)	Quartile 2 (n = 525) (5.10–9.39 *μ*g/ml)	Quartile 3 (n = 518) (9.40–16.79 *μ*g/ml)	Quartile 4 (n = 519) (16.80–79.88 *μ*g/ml)	
Age (year)	52.3 ± 12.0	53.5 ± 12.0	54.2 ± 12.4	54.1 ± 12.4	0.039
Female (%)	120 (22.9)	185 (35.2)	224 (43.2)	285 (54.9)	<0.001
SBP (mmHg)	127.4 ± 15.6	129.0 ± 15.5	127.6 ± 16.4	129.8 ± 18.1	0.065
DBP (mmHg)	76.7 ± 11.2	77.0 ± 10.8	76.6 ± 10.5	77.1 ± 11.9	0.888
Current/former Smoking (%)	297 (56.6)	258 (49.2)	225 (43.5)	195 (37.6)	<0.001
DM (%)	217 (41.3)	190 (36.2)	173 (33.4)	202 (38.9)	0.049
HbA1c (%)	6.8 ± 1.5	6.7 ± 1.3	6.7 ± 1.3	6.7 ± 1.5	0.825
HTN (%)	510 (97.1)	513 (97.7)	492 (95.0)	507 (97.7)	0.034
BMI (kg/m^2^)	25.5 ± 3.2	25.0 ± 3.3	24.3 ± 3.4	23.4 ± 3.3	<0.001
Creatinine (mg/dl)	1.6 ± 0.8	1.6 ± 0.9	1.8 ± 1.1	2.3 ± 1.5	<0.001
eGFR (ml/min/1.73 m^2^)	62.1 ± 31.0	58.4 ± 30.3	50.1 ± 28.5	41.9 ± 29.3	<0.001
hsCRP (mg/L)	2.5 ± 5.9	1.8 ± 4.6	2.1 ± 5.7	1.6 ± 4.4	0.027
Albumin (g/dl)	4.3 ± 0.3	4.2 ± 0.4	4.2 ± 0.4	4.0 ± 0.5	<0.001
UACR (mg/g)	559.7 [1.3–6688.1]	757.8 [0.7–7967.2]	924.1 [1.6–8559.2]	1361.8 [2.2–12586.9]	<0.001
LDL (mg/dl)	93.0 [21.0–225.0]	92.2 [24.8–206.0]	94.0 [29.0–242.0]	93.0 [33.0–273.0]	0.541
HDL (mg/dl)	44.5 ± 12.4	47.4 ± 14.4	51.1 ± 15.9	54.6 ± 16.8	<0.001
TG (mg/dl)	186.0 ± 118.1	167.7 ± 99.2	148.4 ± 90.6	127.4 ± 75.6	<0.001
ACEi (%)	52 (9.9)	67 (12.8)	47 (9.1)	68 (13.1)	0.093
ARB (%)	423 (80.6)	427 (81.3)	403 (77.8)	414 (79.8)	0.523

*Abbreviations*: SBP, systolic blood pressure; DBP, diastolic blood pressure; DM, diabetes mellitus; HbA1c, hemoglobin A1c; HTN, hypertension; BMI, body mass index; eGFR, estimated glomerular filtration rate; hsCRP, high-sensitivity C-reactive protein; UACR, urine albumin-to-creatinine ratio; LDL, low-density lipoprotein; HDL, high-density lipoprotein; TG, triglyceride; ACEi, angiotensin converting enzyme inhibitor; ARB, angiotensin receptor blocker.

### eGFR and urine albumin-to-creatinine ratio according to serum adiponectin levels

**Figure 1:**
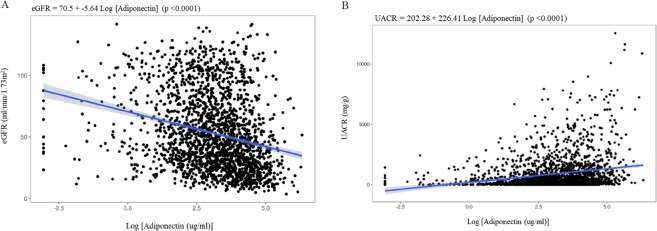
Correlation between serum adiponectin levels and eGFR (**A**) and UACR (**B**). The shaded region around the blue lines represents the 95% confidence interval. eGFR, estimated glomerular filtration rate; UACR, urine albumin-creatinine ratio.

Higher serum adiponectin levels were significantly associated with greater age, female sex, non-smoking status, hypertension, higher serum creatinine level, higher urine albumin-to-creatinine ratio (UACR), lower body mass index (BMI), lower high-sensitivity C-reactive protein (hsCRP), and lower albumin. Furthermore, the serum adiponectin level exhibited an inverse correlation with eGFR (p < 0.001) (Fig. 1A) and a positive correlation with UACR (p < 0.001) (Fig. 1B).

### Association between serum adiponectin quartiles and composite renal outcome

**Figure 2:**
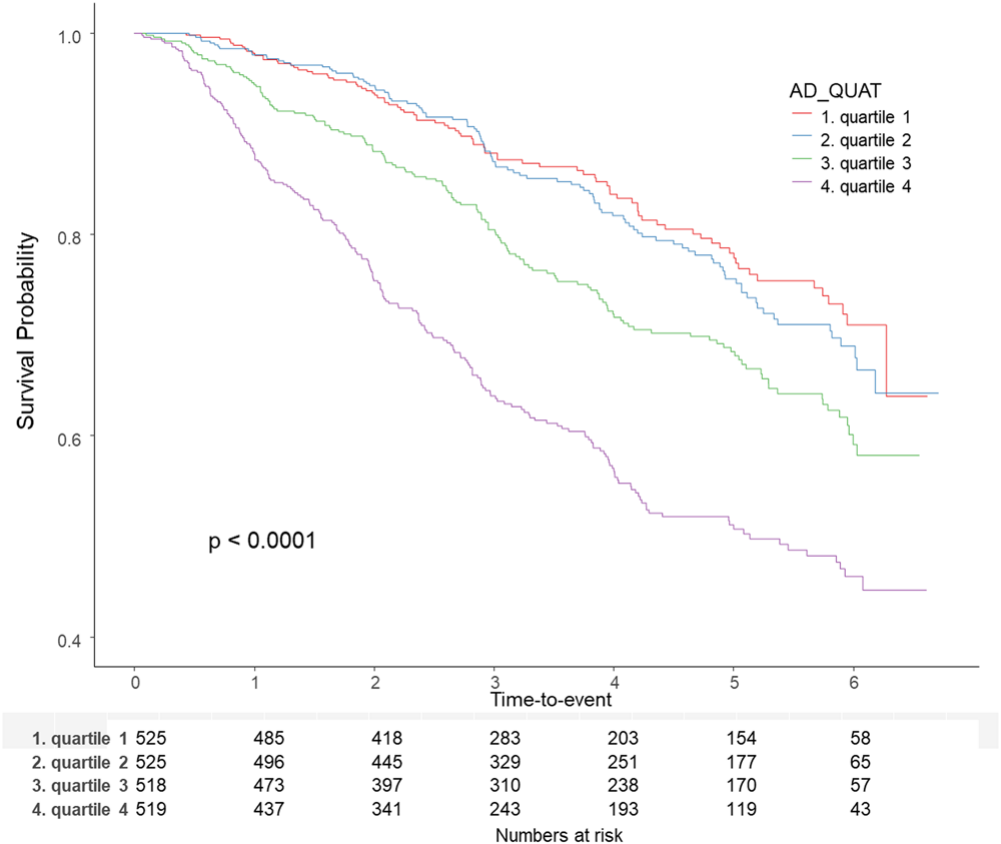
Kaplan-Meier survival curves showed that higher quartile is associated poor renal outcome.

A total of 566 (25.2%) patients experienced a composite renal outcome during the follow-up period. Adverse renal outcomes occurred in 87 (16.6%), 108 (20.6%), 150 (29.0%), and 221 (42.6%) patients in quartiles 1, 2, 3, and 4, respectively (p < 0.001). On unadjusted analysis, the HR of serum adiponectin was 1.04 (95% CI, 1.03 to 1.04; p < 0.001), and the HR of the highest quartile was 2.92 (95% CI, 2.28 to 3.74; p < 0.001). The Kaplan-Meier survival curves (Fig. 2) indicate that the higher quartile was associated with poor renal outcome (p < 0.001).

In order to quantify the correlation between adiponectin and covariates, we performed multivariable analysis (Table 2). In model 1, patients in the highest quartile had a significantly higher risk of renal outcome than those in the lowest quartile after adjustment for age and sex (HR, 3.13; 95% CI, 2.42 to 4.05; p < 0.001). In model 2, we added systolic blood pressure (BP), DM, smoking, BMI, LDL, serum albumin, UACR, and drug history of angiotensin converting enzyme inhibitors (ACEi) or angiotensin receptor blockers (ARB). The HR of the highest quartile was 2.29 (95% CI, 1.73 to 3.04; p < 0.001). In a fully adjusted model that added eGFR (model 3), the highest adiponectin quartile was associated with an increased risk of composite renal outcome (HR, 1.39; 95% CI, 1.05–1.84; p = 0.021). In model 3, the risk of composite renal outcome increased by 1% (HR, 1.01; 95% CI, 1.00–1.02; p = 0.029) for every 1 µg/ml increase in baseline adiponectin levels. We also performed univariable analysis for the covariates (Supplementary Table S1).

**Table 2:** HRs (95% CIs) for composite renal outcome according to adiponectin quartile.

	Unadjusted		Adjusted					
			Model 1		Model 2		Model 3	
	HR (95% CI)	p-value	HR (95% CI)	p-value	HR (95% CI)	p-value	HR (95% CI)	p-value
Serum adiponectin (µg/ml)	1.04 (1.03–1.04)	<0.001	1.04 (1.03–1.05)	<0.001	1.02 (1.02–1.03)	<0.001	1.01 (1.00–1.02)	0.029
Quartile 1	1 (reference)	—	1 (reference)	—	1 (reference)	—	1 (reference)	—
Quartile 2	1.12 (0.85–1.49)	0.427	1.14 (0.86–1.52)	0.351	0.97 (0.72–1.30)	0.827	0.85 (0.63–1.13)	0.262
Quartile 3	1.65 (1.27–2.15)	<0.001	1.71 (1.31–2.24)	<0.001	1.53 (1.16–2.01)	0.003	1.08 (0.82–1.43)	0.571
Quartile 4	2.92 (2.28–3.74)	<0.001	3.13 (2.42–4.05)	<0.001	2.29 (1.73–3.04)	<0.001	1.39 (1.05–1.84)	0.021

Model 1: adjusted for age and sex.

Model 2: Model 1 + SBP, DM, smoking, BMI, LDL, serum albumin, UACR and ACEi or ARB.

Model 3: Model 2 + eGFR.

**Figure 3:**
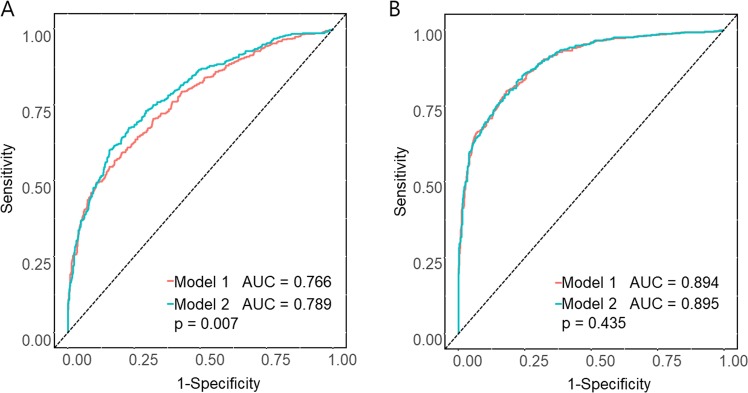
ROC curves for 5 year renal outcome. At (**A**), model 1 included age, sex, SBP, DM, smoking, BMI, LDL, serum albumin, UACR and ACEi or ARB for renal outcome. Model 2 included model 1 + adiponectin quartiles. P-value for AUC difference between model 1 and 2 was 0.007. At (**B**), model 1 and model 2 included eGFR at (**A**) P-value for AUC difference was 0.435.

After introducing the eGFR into model 2 of Table 2, the HR dropped from 2.29 to 1.39. It was difficult to determine if serum adiponectin is an independent risk factor of renal outcome. So we performed time dependent receiver operating characteristic (ROC) curve analysis (Fig. 3). At Fig. 3A, model 1 included age, sex, SBP, DM, smoking, BMI, LDL, serum albumin, UACR and ACEi or ARB for renal outcome. Model 2 included model 1 + adiponectin quartiles. P-value for AUC difference between model 1 and 2 was 0.007. At Fig. 3B, model 1 and model 2 included eGFR at Fig. 3A. Unlike Fig. 3A, there was no significant predictive accuracy of serum adiponectin for renal outcome (p = 0.435) if eGFR was included. These findings mean that serum adiponectin has a predictive accuracy for renal outcome considering multiple confounders except eGFR. But if considering eGFR, the power of adiponectin as predictor is lost.

### Subgroup analysis

**Figure 4:**
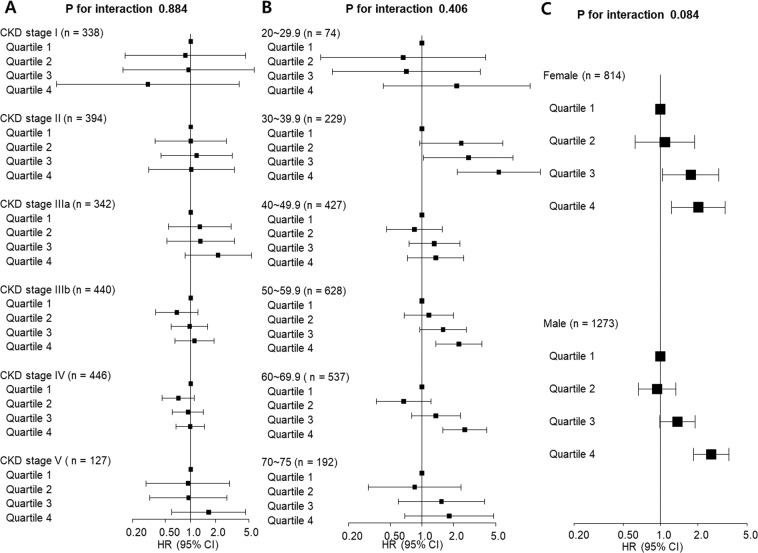
Subgroup analysis for the association between adiponectin level and composite renal outcome according to CKD stage (**A**), age (**B**), or sex (**C**). Central markers mean hazard ratio (HR) and the error whiskers mean 95% confidence interval (CI) of composite renal outcome. Adjusted for systolic blood pressure, diabetes mellitus, serum albumin and urine albumin-creatinine ratio.

We further analysed the association between the serum adiponectin level and composite renal outcomes in several subgroups (Fig. 4). There were no significant effect modification by CKD stage, age, or sex subgroups.

## Discussion

In this study, we demonstrated that an increased serum adiponectin level was significantly associated with old age, female sex, hypertension, higher serum creatinine level, higher UACR, lower BMI, reduced eGFR, and lower serum albumin level. The highest serum adiponectin quartile was also associated with an increased risk of adverse renal outcomes on multivariable analysis in a pre-dialysis CKD cohort.

The mechanisms that underly the relationship between high serum adiponectin and kidney disease progression have not been fully understood. Adiponectin, a vasoactive adipokine, circulates as a trimer, hexamer, or higher-molecular mass form in blood^18,19^. Adiponectin functions by activating the adenosine monophosphate-activated protein kinase (AMPK) pathway^20^ via the AdipoR receptors, including AdipoR1 and AdipoR2. The AMPK pathway is necessary for the maintenance of normal renal physiology^21^. AMPK normally inhibits protein synthesis and its suppression appears to facilitate high-glucose-induced protein synthesis. AMPK is also a potent oxidative stress inhibitor, controlling the generation of reactive oxygen species^22^. These data indicate that adiponectin could potentially contribute to the maintenance of normal renal function. Adiponectin has anti-inflammatory and insulin-sensitizing properties, as well as cardio-protective effects on endothelial cells^23^.

However, in this study, high adiponectin level was associated with an increased risk of adverse renal outcomes. In view of the clearance of serum adiponectin via glomerular filtration, higher serum adiponectin may be observed in CKD patients due to impaired urinary excretion^24^. Moreover, adiponectin levels decrease after kidney transplantation, implying that the kidneys play an important role in the biodegradation or elimination of this protein^25^.

In this study, higher adiponectin was associated with an increased risk of adverse renal outcomes in pre-dialysis CKD patients after adjusting for accepted risk factors including old age, diabetes, hypertension, smoking, and a sedentary lifestyle. However, after adjusting for the eGFR, the power of adiponectin as a predictor of renal outcomes, disappeared. We speculate that serum adiponectin is biomarker of renal dysfunction rather than a true risk factor, intimately involved in CKD progression. However, further investigation is needed to identify the critical position of adiponectin in the pathogenesis of CKD.

This study has several limitations. Lo *et al*. reported that the ratio between high- and low-molecular-weight adiponectin may be an important biomarker of high cardiovascular risk^26^. Since we measured total serum adiponectin, the individual ratios could not be determined. Moreover, we did not measure adiponectin levels on follow-up appointments. However, this prospective study has certain strengths including a large sample size, comprehensive CKD presentation, and long-term follow-up with regard to disease outcomes.

High serum adiponectin levels are associated with composite renal outcomes in CKD patients. These findings suggest that adiponectin is a biomarker for CKD progression. We, therefore, propose that serum adiponectin may be used as a biomarker of renal disease outcomes.

## Materials and methods

### Study design and population

The KoreaN cohort Study for Outcomes in patients With Chronic Kidney Disease (KNOW-CKD) study, launched in 2011, was a patient-based cohort study that enrolled ethnic Korean adults with CKD. Patients aged between 20 and 75 years with various causes of CKD were initially screened for the study. Data were collected by a well-trained study coordinator using a standardized case report form and protocol. The detailed design and methods of KNOW-CKD have been published previously^27^.

All procedures performed in studies involving human participants were in accordance with the ethical standards of the institutional and national research committees of the participating institute (IRB approval number NCT01630486) and with the 1964 Helsinki declaration and its later amendments or comparable ethical standards. The study protocol was approved in 2011 by the institutional review board of each participating clinical centre including the Seoul National University Hospital, Seoul National University Bundang Hospital, Severance Hospital, Kangbuk Samsung Medical Center, Seoul St. Mary’s Hospital, Gil Hospital, Eulji General Hospital, Chonnam National University Hospital, and Pusan Paik Hospital. All participating patients provided written informed consent.

A total of 2341 patients with CKD stages ranging from 1 to 5 (predialysis) between April 2011 and February 2016, who voluntarily provided informed consent, were recruited. Among them, 103, who did not satisfy the inclusion criteria or did not have data of isotope dilution mass spectrometry (IDMS)-calibrated creatinine were excluded, leaving 2238 participants. We additionally excluded subjects with missing data for adiponectin and renal outcome; 2087 patients were finally analysed. The mean length of follow-up is 3.56 years.

### Data collection

Baseline socio-demographic data including smoking history, anthropometric measurement, medications, and comorbidities were obtained from the electronic data management system developed by the Seoul National University Medical Research Collaborating Center. Blood samples were obtained in serum separation tubes, centrifuged within 1 h for serum separation, and were sent to the central laboratory of the KNOW-CKD study (Lab Genomics, Seongnam, Republic of Korea). Laboratory tests including complete blood count and blood chemistries were performed at baseline and at 6 months for the first year, and then annually thereafter. Serum creatinine concentrations were measured using an isotope-dilution mass spectrometry-traceable method. Total serum adiponectin concentrations were measured using a commercially available ELISA kit (Adipogen, Incheon, Korea), with intra- and inter-assay coefficients of variations of <3.84 and <5.50%, respectively. Samples were assayed in duplicate, and all results were reported as mean values. eGFR was calculated using the CKD Epidemiology Collaboration equation^28^.

### Primary outcome

Composite renal outcome was defined as one or more of the following: beginning of dialysis or transplantation, a two-fold increment in baseline serum creatinine levels, or a 50% decrease in the eGFR during the follow-up period. A Cox proportional hazard ratio model was applied to analyse the relationship between composite renal outcomes and serum adiponectin levels.

### Statistical analyses

The enrolled patients were divided into quartiles according to their serum adiponectin levels. demographic and biochemical characteristics of the study population according to serum adiponectin level quartiles were compared using the Pearson Chi-square test for categorical variables. One-way analysis of variance was used for normally distributed data, and the Kruskal–Wallis test was used for skewed data to identify differences and compare clinical characteristics between the groups. The Pearson’s correlation test was used to evaluate the relationship between covariables, and a linear regression model was constructed after adjustment for multiple confounders. The Cox proportional hazard regression model was applied to survey the independent risk factors associated with composite renal outcome. Hazard ratios (HR) and 95% confidence intervals (CI) were calculated to compare the risk of composite renal outcome. The analyses were performed using R language (version 3.4.4; R Foundation for Statistical Computing).

## Supplementary information

Supplementary Table S1

## Data Availability

Available as supplementary material when accepted.
