# Supplementary material for: High serum adiponectin as a biomarker of renal dysfunction: Results from the KNOW-CKD study
Source: Sci Rep. 2020 Mar 27;10:5598. doi: 10.1038/s41598-020-62465-2 (PMC7101406; doi:10.1038/s41598-020-62465-2)
Supplement: Supplementary file 1 — Supplementary Table S1 [file 41598_2020_62465_MOESM1_ESM.docx]

**High serum adiponectin is associated with chronic kidney disease progression: Results from the KNOW-CKD study**

Su Hyun Song, M.D.^1^,Tae Ryom Oh, M.D.^1^, Hong Sang Choi, M.D.^1^, Chang Seong Kim, M.D., Ph.D.^1^, Seong Kwon Ma, M.D., Ph.D.^1^, Kook Hwan Oh, M.D., Ph.D.^2^ Curie Ahn, M.D., Ph.D.^2^ _,_ Soo Wan Kim, M.D., Ph.D.^1^* and Eun Hui Bae, M.D., Ph.D.^1^*

*^1^Department of Internal Medicine, Chonnam National University Medical School, Gwangju, Korea; and ^2^Department of Internal Medicine, Seoul National University, Seoul, Korea*

^*^These authors have contributed equally to this manuscript as correspondence authors.

**Running Title:** Serum adiponectin in CKD progression

**Correspondence to:** Eun Hui Bae or Soo Wan Kim

Department of Internal Medicine, Chonnam National University Medical School,

42 Jebongro, Gwangju, 61469, Korea

E-mail: baedak76@gmail.com or skimw@chonnam.ac.kr

Phone: +82-62-220-6503 or +82-62-220-6271

**Table S1. HRs (95% CIs) for composite renal outcome in univariable analysis according to serum adiponectin quartiles**

| Covariates | Quartile 1 | Quartile 2 | | Quartile 3 | | Quartile 4 | |
| --- | --- | --- | --- | --- | --- | --- | --- |
|  | HR  (95% CI) | HR  (95% CI) | p-value | HR  (95% CI) | p-value | HR  (95% CI) | p-value |
| Age | 1 (reference) | 1.11  (0.84-1.47) | 0.470 | 1.63  (1.25-2.12) | <0.001 | 2.88  (2.24-3.69) | <0.001 |
| Sex | 1 (reference) | 1.16  (0.87-1.54) | 0.314 | 1.74  (1.33-2.27) | <0.001 | 3.18  (2.46-4.11) | <0.001 |
| Systolic BP | 1 (reference) | 1.10  (0.83-1.46) | 0.520 | 1.70  (1.30-2.21) | <0.001 | 2.87  (2.24-3.69) | <0.001 |
| DM | 1 (reference) | 1.18  (0.89-1.56) | 0.256 | 1.83  (1.40-2.39) | <0.001 | 3.18  (2.48-4.08) | <0.001 |
| Smoking | 1 (reference) | 1.15  (0.87-1.52) | 0.336 | 1.73  (1.33-2.26) | <0.001 | 3.15  (2.44-4.05) | <0.001 |
| BMI | 1 (reference) | 1.14  (0.86-1.51) | 0.377 | 1.74  (1.33-2.27) | <0.001 | 3.21  (2.49-4.14) | <0.001 |
| LDL | 1 (reference) | 1.12  (0.84-1.50) | 0.423 | 1.68  (1.28-2.19) | <0.001 | 2.99  (2.33-3.84) | <0.001 |
| Serum albumin | 1 (reference) | 0.98  (0.73-1.30) | 0.867 | 1.45  (1.11-1.89) | 0.007 | 1.99  (1.53-2.58) | <0.001 |
| UACR | 1 (reference) | 1.00  (0.75-1.33) | 0.994 | 1.41  (1.08-1.85) | 0.012 | 2.06  (1.59-2.68) | <0.001 |
| eGFR | 1 (reference) | 0.94  (0.71-1.25) | 0.680 | 1.12  (0.86-1.46) | 0.407 | 1.55  (1.20-1.99) | <0.001 |

**The author details**

**Su Hyun Song, M.D**.^1^: Fellow-Division of nephrology, Department of internal medicine, Chonnam National University Medical School, Gwangju, Korea

**Tae Ryom Oh, M.D**.^1^: assistant professor- Division of nephrology, Department of internal medicine, Chonnam National University Medical School, Gwangju, Korea

**Hong Sang Choi**, M.D.^1^: assistant professor- Division of nephrology, Department of internal medicine, Chonnam National University Medical School, Gwangju, Korea

**Chang Seong Kim,** M.D., Ph.D.^1^: assistant professor- Division of nephrology, Department of internal medicine, Chonnam National University Medical School, Gwangju, Korea

**Seong Kwon Ma,** M.D., Ph.D.^1^: professor- Division of nephrology, Department of internal medicine, Chonnam National University Medical School, Gwangju, Korea

**Kook Hwan Oh**, M.D., Ph.D.^2^ : professor- Division of nephrology, Department of internal medicine, Seoul National University Medical School, Seoul, Korea

**Curie Ahn,** M.D., Ph.D.^2^ :professor- Division of nephrology, Department of internal medicine, Seoul National University Medical School, Seoul, Korea

**Soo Wan Kim**, M.D., Ph.D.^1^*: professor- Division of nephrology, Department of internal medicine, Chonnam National University Medical School, Gwangju, Korea

**Eun Hui Bae**, M.D., Ph.D.^1^*: professor- Division of nephrology, Department of internal medicine, Chonnam National University Medical School, Gwangju, Korea
